# The complete chloroplast genome of *Oxytropis monophylla* (Fabaceae): insights into genome features and evolutionary relationships

**DOI:** 10.1080/23802359.2025.2548846

**Published:** 2025-08-18

**Authors:** Chaopan Zhang, Shenglin Zhao, Almira Ablat, Xiaodong Ding, Wenchao Hu

**Affiliations:** a Key Laboratory of Ecological Protection of Agro-pastoral Ecotones in the Yellow River Basin National Ethnic Affairs Commission of the People’s Republic of China, School of Biological Science & Engineering, North Minzu University, Yinchuan, Ningxia, PR China; b Agriculture and Animal Husbandry Comprehensive Administrative Law Enforcement Brigade of Alashan Left Banner, Alxa League, Inner Mongolia, PR China

**Keywords:** Oxytropis monophylla, Fabaceae, chloroplast genome, inverted repeat regions loss, phylogenetic analysis

## Abstract

*Oxytropis monophylla* belongs to the genus Oxytropis of the family Fabaceae, and is an ecologically significant species, as it plays a vital role in desertification control. However, the chloroplast genome of *O. monophylla* remains unreported. Here, we constructed a high-quality chloroplast (cp) genome assembly for *O. monophylla* using NOVOPlasty and Illumina sequencing data. The results showed that the *O. monophylla* cp genome exhibited atypical structural characteristics due to the absence of an IR region. The chloroplast genome was 122,413 bp long with a GC content of 34.2%, encoding 108 complete genes. Phylogenetic reconstruction through both maximum likelihood and Bayesian inference methods resolved 12 *Oxytropis* species into two strongly supported clades (BS, PP =100%, 1). The *O. monophylla* and *O. bicolor* formed a monophyletic group, indicates a close relationship between the two species. The first chloroplast genome of *O. monophylla* establishes a good foundation for further genetic and genomic studies of the *Oxytropis* genus.

## 1. Introduction

Chloroplast are essential organelles that are central participants in plant cells and involved in photo-synthesis and carbon fixation (Li et al. 2015; Hollingsworth et al. 2016). In most plants, the chloroplast genome is inherited, unlike the nuclear genome, which facilitates the inference of species delimitation, genetic diversity, and evolution (Zhang et al. 2012; Daniell et al. 2016; Guo et al. 2017; Zhang et al. 2018; Xie et al. 2022; Ran et al. 2024). Recent advancements in sequencing technology have enabled the publication of numerous chloroplast genomes across various plant taxa, enabling large-scale comparative analyses across diverse plant lineages (Hu et al. 2016; Zhang et al. 2018; Yang et al. 2022).

*Oxytropis monophylla* Grubov (Grubov 1978) belongs to the genus *Oxytropis* of the family Fabaceae, is a perennial herbaceous species endemic to semi-desert regions of Ningxia and Inner Mongolia in northern China (Malyshev 2008; Zhu et al. 2010). As an ecologically significant species, it plays a vital role in desertification control through its remarkable sand-binding capacity, attributed to its well-developed root system and robust vegetative propagation (Wang et al. 2021). In addition to ecological services, *O. monophylla* also plays an important role as a feed resources. Recent studies reveal its high nutritional value, containing 15%–18% protein and substantial fiber content, making it a preferred early spring feed for ruminants in arid regions (Li et al. 2015; Bei et al. 2022). Despite these important attributes, the species remains understudied compared to other Fabaceae members. In particular, the chloroplast genome of *O. monophylla* remains unreported.

In this study, we assembled and analyzed the complete chloroplast genome of *O. monophylla* for the first time. Our aims were (1) to characterize the structural features of the CPGs for the *O. monophylla*, and (2) to resolve the evolutionary relationships of *O. monophylla*, and to provide data support for the species identification and phylogenetic relationship of *Oxytropis*.

## 2. Materials

**Figure 1.**
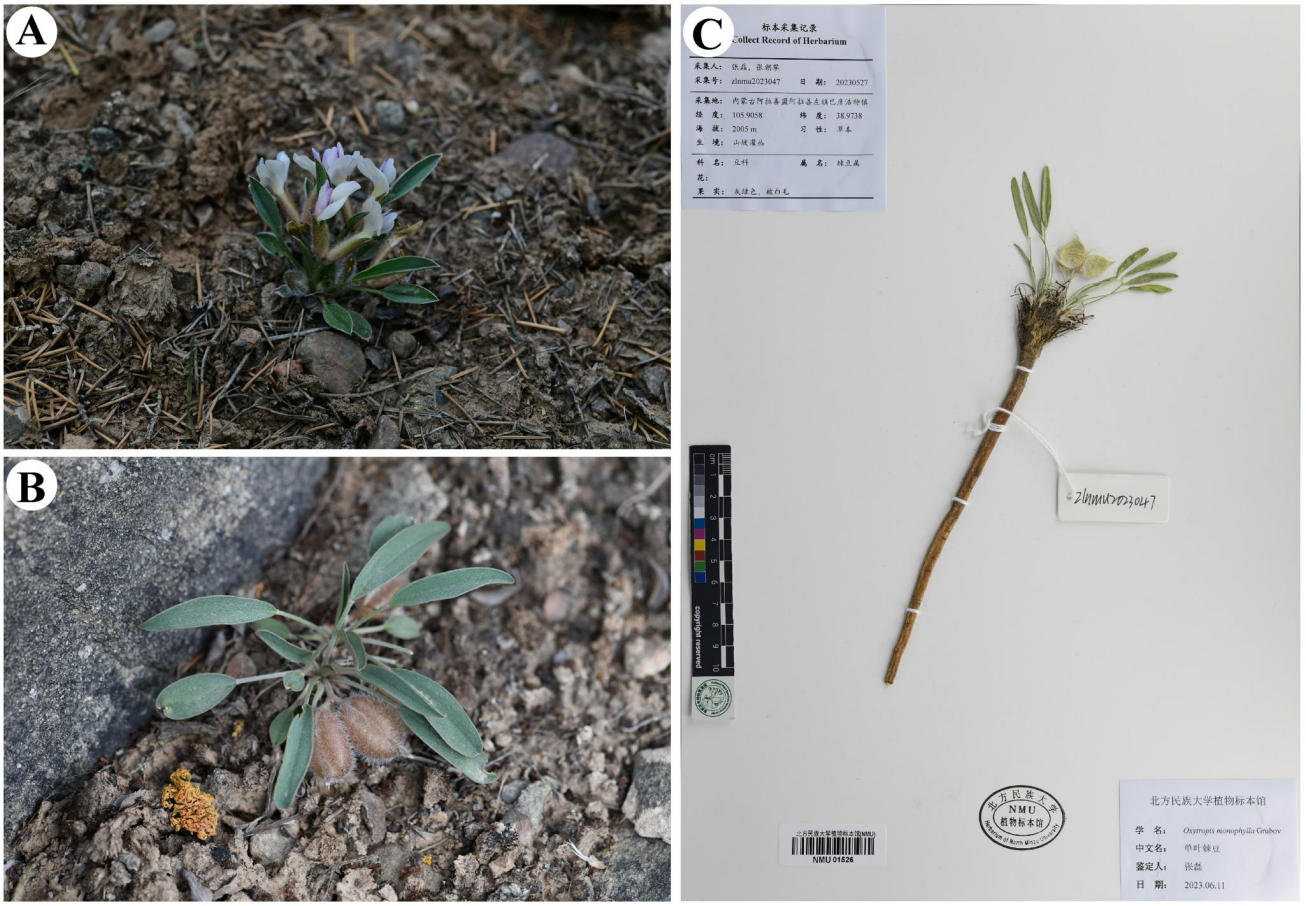
The photos of *O. monophylla* (photographed by Chaopan Zhang). (A) Whole plant and flowers. (B) Whole plant and fruits. (C) Herbarium of *O. monophylla* (petiole 0.5–1 cm; legume sessile, pubescent, ovoid).

Healthy fresh leaves of *O. monophylla* were collected from Bayanhot of Alxa Left Banner (Alxa League, Inner Mongolia, China; coordinates: 105.9058E, 38.9738 N) (by Chaopan Zhang: 3024891762@qq.com) (Figure 1), and immediately desiccated using silica gel. Whole plants with reproductive structures (flowers or fruits) were additionally collected for voucher specimen preparation. The voucher specimen (accession number: zlnmu2023047) was deposited in the Herbarium of North Minzu University.

## 3. Methods

Total genomic DNA was isolated with a modified CTAB method (Doyle and Doyle 1987). Sequencing libraries were prepared with the NEBNext DNA Library Kit, during which genomic DNA was fragmented into 350 bp. High-throughput sequencing was subsequently carried out on the Illumina NovaSeq 6000 platform with 150 bp paired-end read length. We acquired 6.2 GB of high-quality data after adapter trimming.

For chloroplast genome assembly, the *de novo* assembly was implemented in NOVOPlasty 4.3 (Dierckxsens et al. 2017) with the specified parameters: k-mer = 39 and genome range 120,000–200,000 bp, using the complete chloroplast genome of *Oxytropis hirta* (PP836294) as a reference. Genome annotation was executed through Plann v1.1 (Huang and Cronk 2015) with subsequent manual verification in Geneious v11.0.3 (Kearse et al. 2012). Sequencing depth analysis was quantified using Samtools (Li et al. 2009).

To establish the phylogenetic placement of *O. monophylla* in *Oxytropis*, the chloroplast genomes of 12 representative species were retrieved from NCBI GenBank to reconstruct the chloroplast genome phylogenetic tree, with *Alhagi sparsifolia* serving as an outgroup. Multiple sequence alignment was performed using MAFFT v.7.313 (Katoh and Standley 2013). Phylogenetic reconstruction employed both Maximum Likelihood (ML) and Bayesian Inference (BI) approaches: ML analysis was conducted in RAxML v8.1.24 (Stamatakis 2014) under the GTR + Γ model, while BI analysis in Mrbayes v 3.2.6 (Ronquist et al. 2012) utilized the GTR+I + G model selected through jModeltest. Final phylogenetic trees were visualized using FigTree v1.4.2 (Rambaut 2012).

## 4. Results

**Figure 2.**
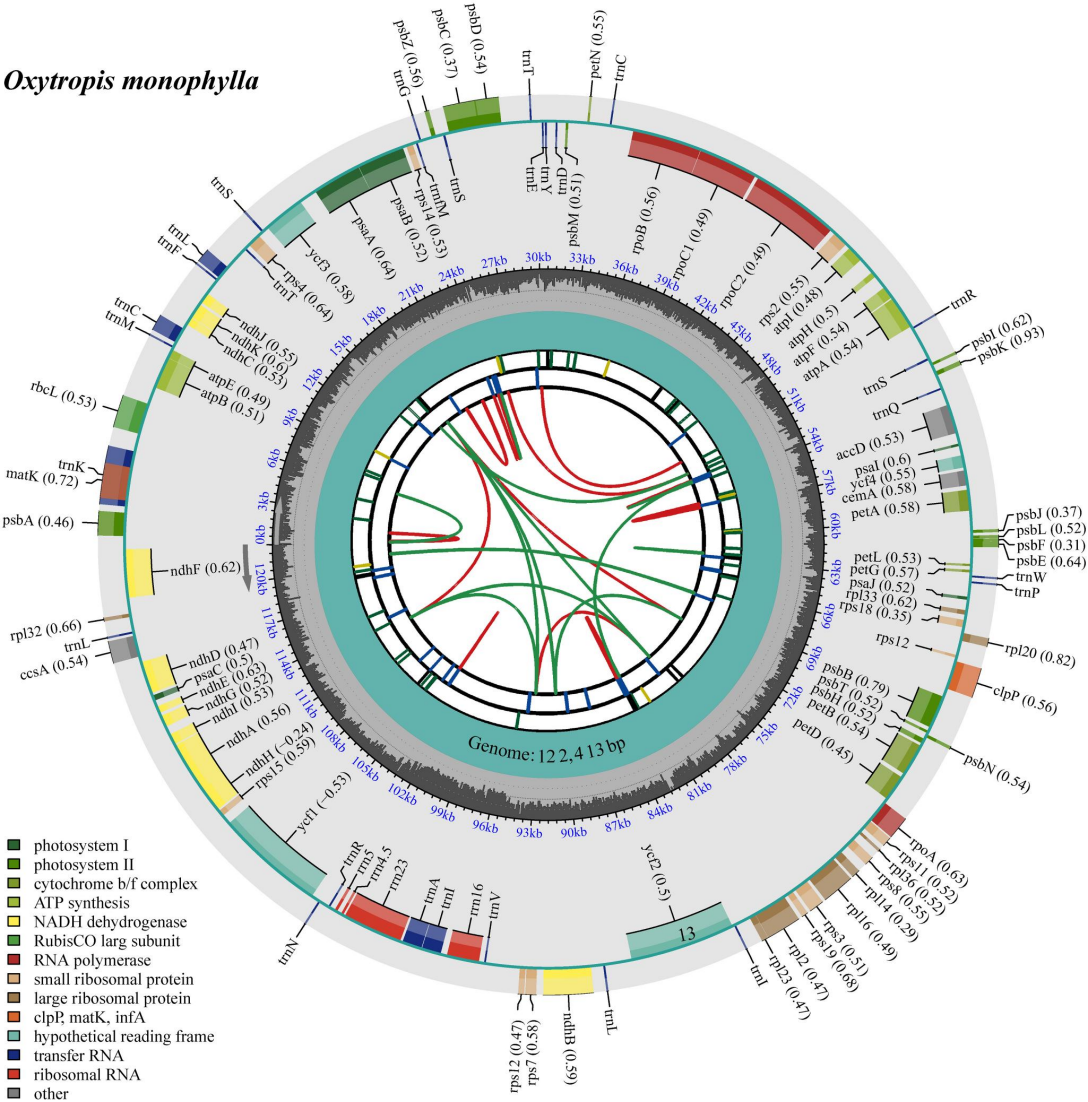
The detailed genome collinearity map of *O. monophylla* cp genome. The species name and GC content are shown in the top left corner. The map contains six tracks in default. From the center outward, the first track shows the dispersed repeats. The dispersed repeats consist of direct and palindromic repeats, connected with red and green arcs. The second track shows the long tandem repeats as short blue bars. The third track shows the short tandem repeats or microsatellite sequences. The fourth track displays the genome length. The fifth track shows the GC content along the genome, while the sixth track sounds the genes. The gene names are followed by optional information about codon usage bias and color-coded based on their functional classification. The inner genes are transcribed clockwise, and the outer genes are transcribed anticlockwise. The functional type of the genes is shown in the bottom left corner.

Following quality filtering and preprocessing, we obtained at least 3.6 gigabases (Gb) of whole-genome sequencing data, which were retained. These clean reads were used to assemble high-quality chloroplast genomes through a reference-guided approach. The total chloroplast genome of *O. monophylla* (PV240322) was 122,413 bp long, with sequencing depth analysis revealing maximal, minimal, and average coverage values of 7961×, 6275.78×, and 1117×, respectively (Fig. S1). The chloroplast genome displayed a unique structural organization, consisting of only a single copy (Figure 2, Fig. S2).

**Figure 3.**
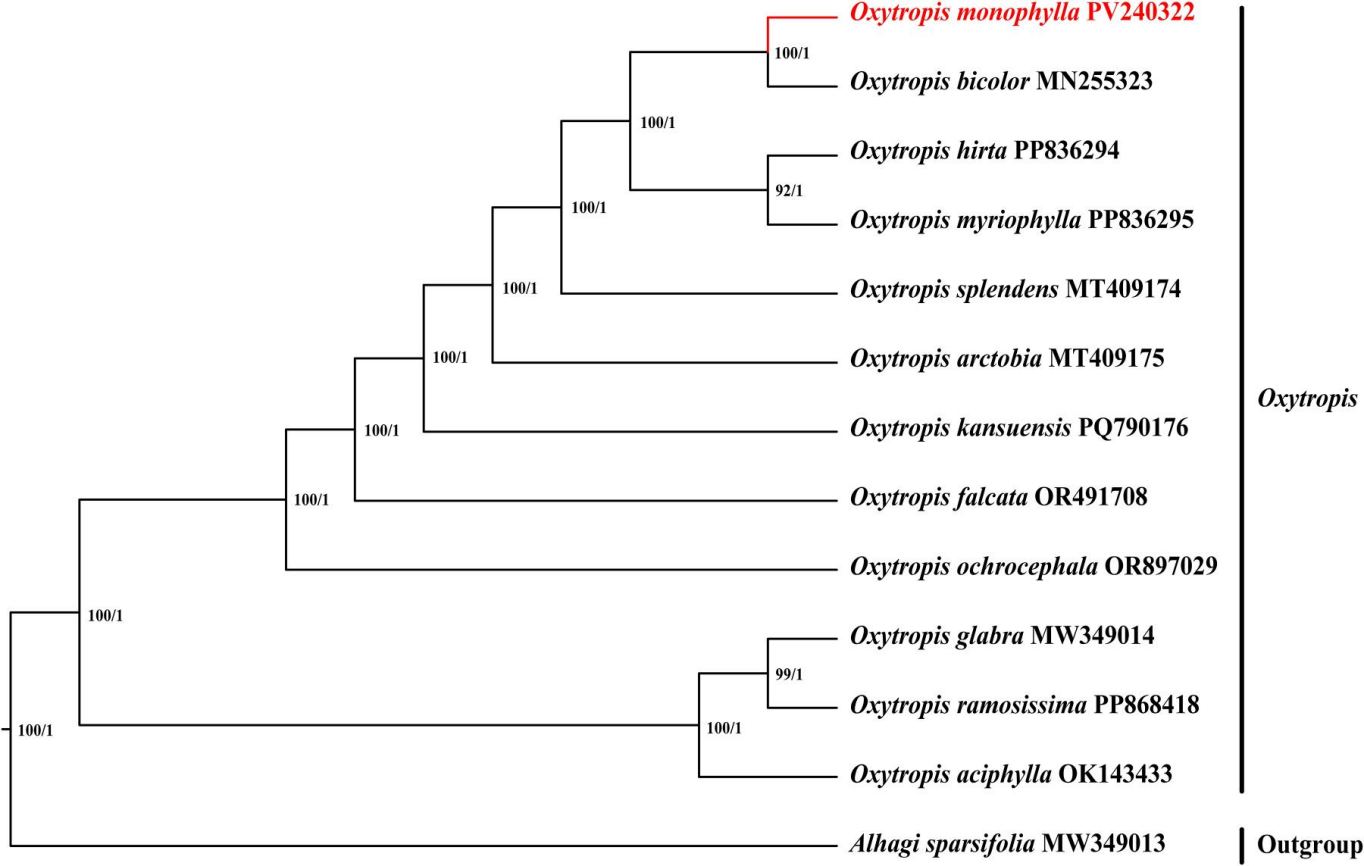
Phylogenetic tree obtained using the maximum likelihood (ML) and bayesian inference (BI) methods of *oxytropis* species based on whole chloroplast genome. GenBank accession numbers: *Aihagi sparsifolia* MW349013 (Jiang et al. 2021), *oxytropis aciphylla* OK143433 (Bei et al. 2022), *oxytropis arctobia* MT409175 (Tavares et al. 2022), *oxytropis bicolor* MN255323 (Su et al. 2019), *oxytropis falcata* OR491708, *oxytropis glabra* MW349014 (Liu et al. 2021), *oxytropis hirta* PP836294, *oxytropis monophylla* (PV240322), *oxyropis kansuensis* PQ790176, *oxytropis myriophylia* PP836295, *oxytropis ochrocephala* OR897029 (Hu et al. 2024), *oxytropis ramosissima* PP868418, *oxyropis splendens* MT409174 (Tavares et al. 2022).

The chloroplast genome of *O. monophylla* annotation identified 108 complete genes (Table S1), comprising 76 protein-coding genes (76 PCGs), 4 ribosomal RNA genes (4 rRNAs), and 28 tRNA genes (28 tRNAs). Additionally, the genome contained 1 trans-splicing gene (Fig. S3) and 9 cis-splicing genes (Fig. S4), with an overall GC content of 34.2%. Phylogenetic reconstruction using both Maximum Likelihood (ML) and Bayesian Inference (BI) methods robustly placed *O. monophylla* within the *Oxytropis* (Figure 3). In these trees, the 12 *Oxytropis* species were divided into two strongly supported monophyletic groups (BS, PP = 100%, 1) (Figure 3). Notably, *O. monophylla* clustered with *O. bicolor* (BS, PP =100%, 1), indicating a close evolutionary relationship between the two species.

## 5. Discussion and conclusion

Previous studies have established that land plant chloroplast genomes generally range in size from 107 to 218 kb (Wicke et al. 2011) and encode 110–130 genes (Zhu et al. 2017). The overall GC content of these genomes falls within 30–45% (Zhu et al. 2017), in which, inverted repeat (IR) regions exhibit higher GC content than large single-copy (LSC) regions, which in turn surpass the GC levels of small single-copy (SSC) regions (Zhang et al. 2012; Daniell et al. 2016). In this study, the complete cp genome of *O. monophylla* was assembled with a total sequence length of 122,413 bp, and 108 complete genes were identified, and the GC content is 34.2%, which is basically consistent with the cp genome characteristics of other species of *Oxytropis* (Su et al. 2019; Liu et al. 2021; Hu et al. 2024). However, the cp genome characteristics of *O. monophylla* are at the smaller end of the spectrum for higher plants organelle genomes. In this study, we observed an intriguing phenomenon, the *O. monophylla* does not display a typical quadripartite structure of higher plants due to the complete absence of inverted repeat (IR) regions. Meanwhile, the characteristic absence of an IR region has also been observed in other cp genomes of *Oxytropis* genus (Su et al. 2019; Liu et al. 2021; Hu et al. 2024). The absence of inverted repeat (IR) regions may represent a conserved genomic feature in chloroplast genomes across the *Oxytropis* genus.

The cp genomes have emerged as a central focus in molecular biology research, and demonstrate significant potential for resolving phylogenetic relationships within angiosperms (Yang et al. 2022; Ran et al. 2024). In this study, phylogenetic analyses using the BI method and the ML method revealed that the cp genomes of *Oxytropis* were divided into two main clades with strong support, and the phylogenetic result was consistent with Liu et al. (2021) and Hu et al. (2024). Notably, *O. monophylla* and *O. bicolor* formed a monophyletic group. To advance our understanding, a comprehensive investigation of *O. monophylla* is necessary, particularly focusing on its codon usage patterns and population-level genomic variations. Such detailed investigations are essential for elucidating structural variations within its chloroplast genome. Furthermore, expanding the collection of *Oxytropis* cp genomes will enhance our comprehension of evolution within this ecologically significant genus.

## Supplementary Material

Supplementary File.docx

## Data Availability

The complete chloroplast genome sequence of *O. monophylla* in this study has been submitted to the NCBI database under the accession number PV240322. The associated BioProject, SRA and BioSample numbers are PRJNA1230366, SRR32570914 and SAMN47182783, respectively.
