# Supplementary material for: The complete chloroplast genome of Oxytropis monophylla (Fabaceae): insights into genome features and evolutionary relationships
Source: Mitochondrial DNA B Resour. 2025 Aug 18;10(9):826–30. doi: 10.1080/23802359.2025.2548846 (PMC12364091; doi:10.1080/23802359.2025.2548846)
Supplement: Supplementary File.docx [file TMDN_A_2548846_SM1460.docx]

The complete chloroplast genome of *Oxytropis monophylla* (Fabaceae): insights into genome features and evolutionary relationships

Chaopan Zhang^1^, Shenglin Zhao^2^, Almira Ablat^1^, Xiaodong Ding^1^, Wenchao Hu^1*^

^1^Key Laboratory of Ecological Protection of Agro-pastoral Ecotones in the Yellow River Basin National Ethnic Affairs Commission of the People’s Republic of China, School of Biological Science & Engineering, North Minzu University, Yinchuan, Ningxia, P. R. China

^2^Agriculture and animal husbandry comprehensive administrative law enforcement brigade of Alashan left banner, Alxa League, Inner Mongolia, P. R. China

***Corresponding author**: Wenchao Hu, huwenchao1985@126.com

**ORCID**: Wenchao Hu; https://orcid.org/0000-0001-8152-4911

Table S1 Genes present in the *Oxytropis monophylla* cp genome

| Group of genes | Name of genes |
| --- | --- |
| Subunits of ATP synthase | *atp*A, *atp*B, *atp*E, *atp*F, *atp*H, *atp*I |
| Subunits of NADH-dehydrogenase | *ndh*A*, *ndh*B*, *ndh*C, *ndh*D, *ndh*E, *ndh*F, *ndh*G, *ndh*H, *ndh*I, *ndh*J, *ndh*K |
| Subunits of cytochrome b/f complex | *pet*A, *pet*B*, *pet*D*, *pet*G, *pet*L, *pet*N |
| Subunits of photosystem I | *psa*A, *psa*B, *psa*C, *psa*I, *psa*J |
| Subunits of photosystem II | *psb*A, *psb*B, *psb*C, *psb*D, *psb*E, *psb*F, *psb*H, *psb*I, *psb*J, *psb*K, *psb*L, *psb*M, *psb*N, *psb*T, *psb*Z |
| Large subunit of ribosome | *rpl*2*, *rpl*14, *rpl*16*, *rpl*20, *rpl*23, *rpl*32, *rpl*33, *rpl*36 |
| Small subunit of ribosome | *rps*2, *rps*3, *rps*4, *rps*7, *rps*8, *rps*11, *rps*12*, *rps*14, *rps*15, *rps*18, *rps*19 |
| DNA dependent RNA polymerase | *rpo*A, *rpo*B, *rpo*C1*, *rpo*C2 |
| Subunit of rubisco | *rbc*L |
| c-type cytochrom synthesis gene | *ccs*A |
| Envelop membrane protein | *cem*A |
| Maturase | *mat*K |
| Protease | *clp*P* |
| Subunit of Acetyl-CoA-carboxylase | *acc*D |
| Conserved open reading frames | *ycf*1, *ycf*2, *ycf*3**, *ycf*4 |
| tRNA | *trn*K-UUU*, *trn*M-CAU, *trn*C-ACA*, *trn*F-GAA, *trn*L-UAA*, *trn*T-UGU, *trn*S-GGA, *trn*fM-CAU, *trn*G-GCC, *trn*S-UGA, *trn*T-GGU, *trn*E-UUC, *trn*Y-GUA, *trn*D-GUC, *trn*C-GCA, *trn*R-UCU, *trn*S-GCU, *trn*Q-UUG, *trn*W-CCA, *trn*P-UGG, *trn*I-CAU, *trn*L-CAA, *trn*V-GAC, *trn*I*, *trn*A-UGC*, *trn*R-ACG, *trn*N-GUU, *trn*L-UAG |
| rRNA | *rrn*4.5, *rrn*5, *rrn*16, *rrn*23 |
| Genes with one or two introns are indicated by one(*) or two asterisks (**), respectively. | |


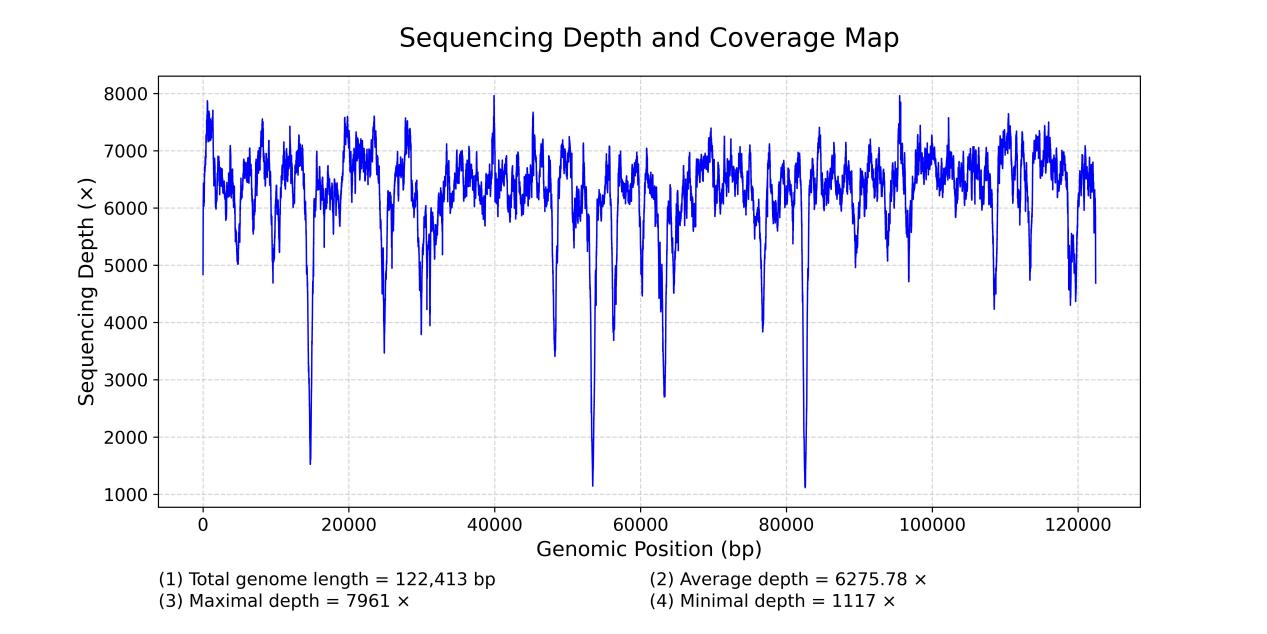


Figure S1 Coverage depth distribution of the *Oxytropis monophylla*.


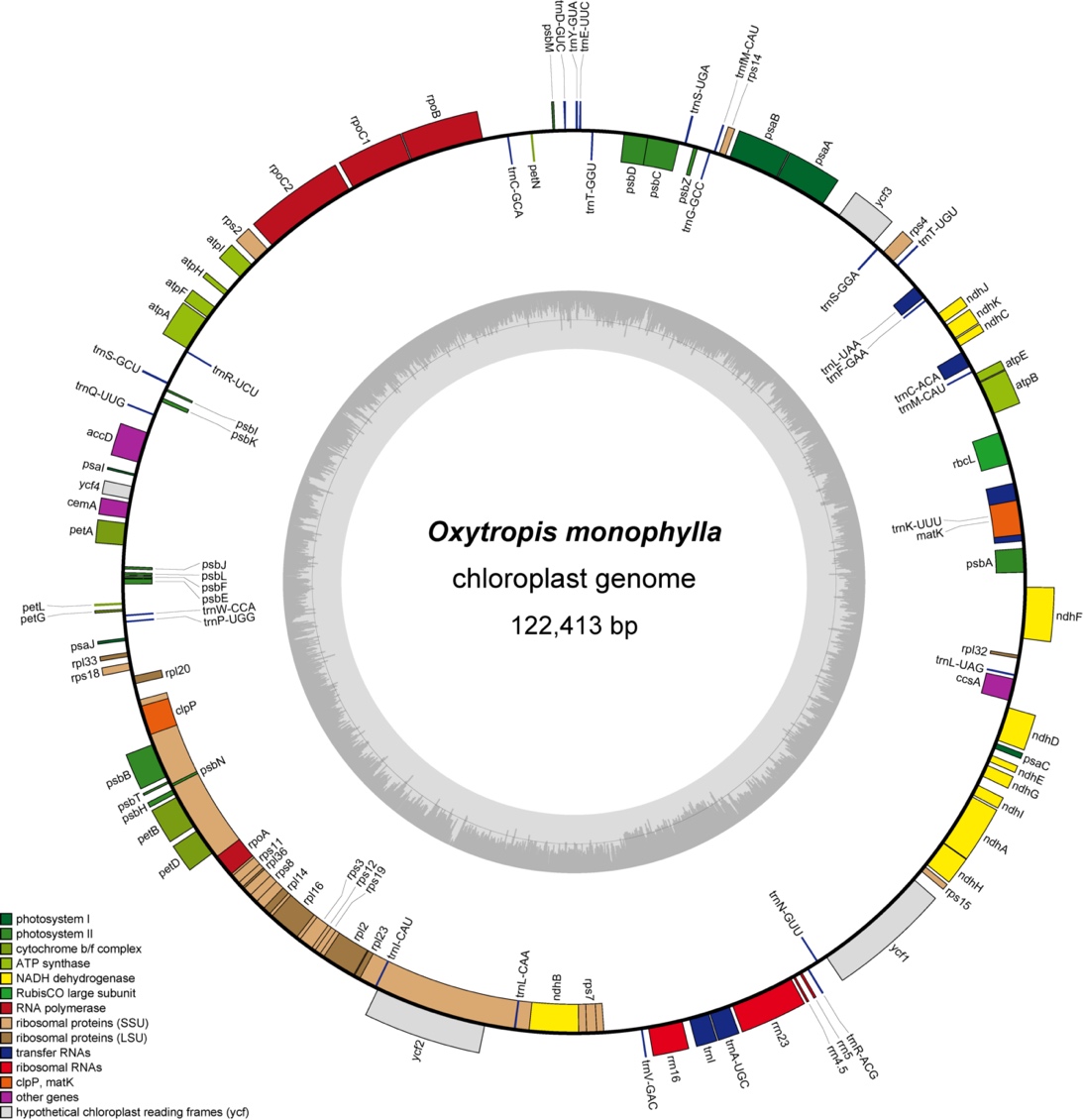


Figure S2 The detailed genome map of *O. monophylla* cp genome. GC content (light gray) is shown in the inside track. Gene models including protein-coding genes, tRNA genes and rRNA genes are shown with various colored boxes in the outer track.


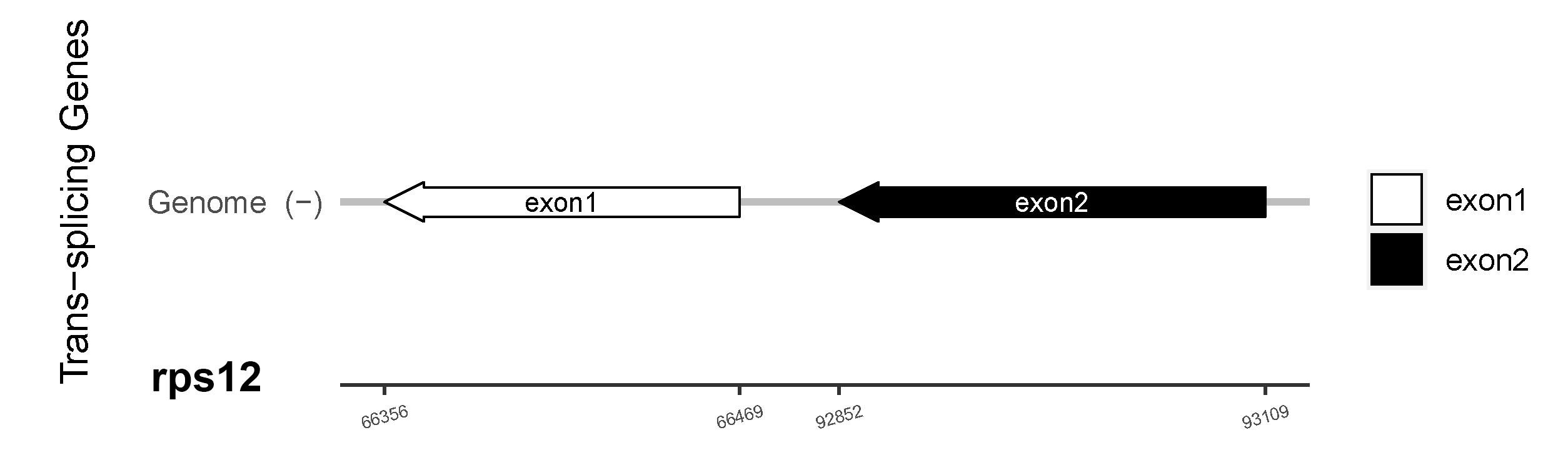


Figure S3 Structure of trans-splicing genes in the *Oxytropis monophylla*.


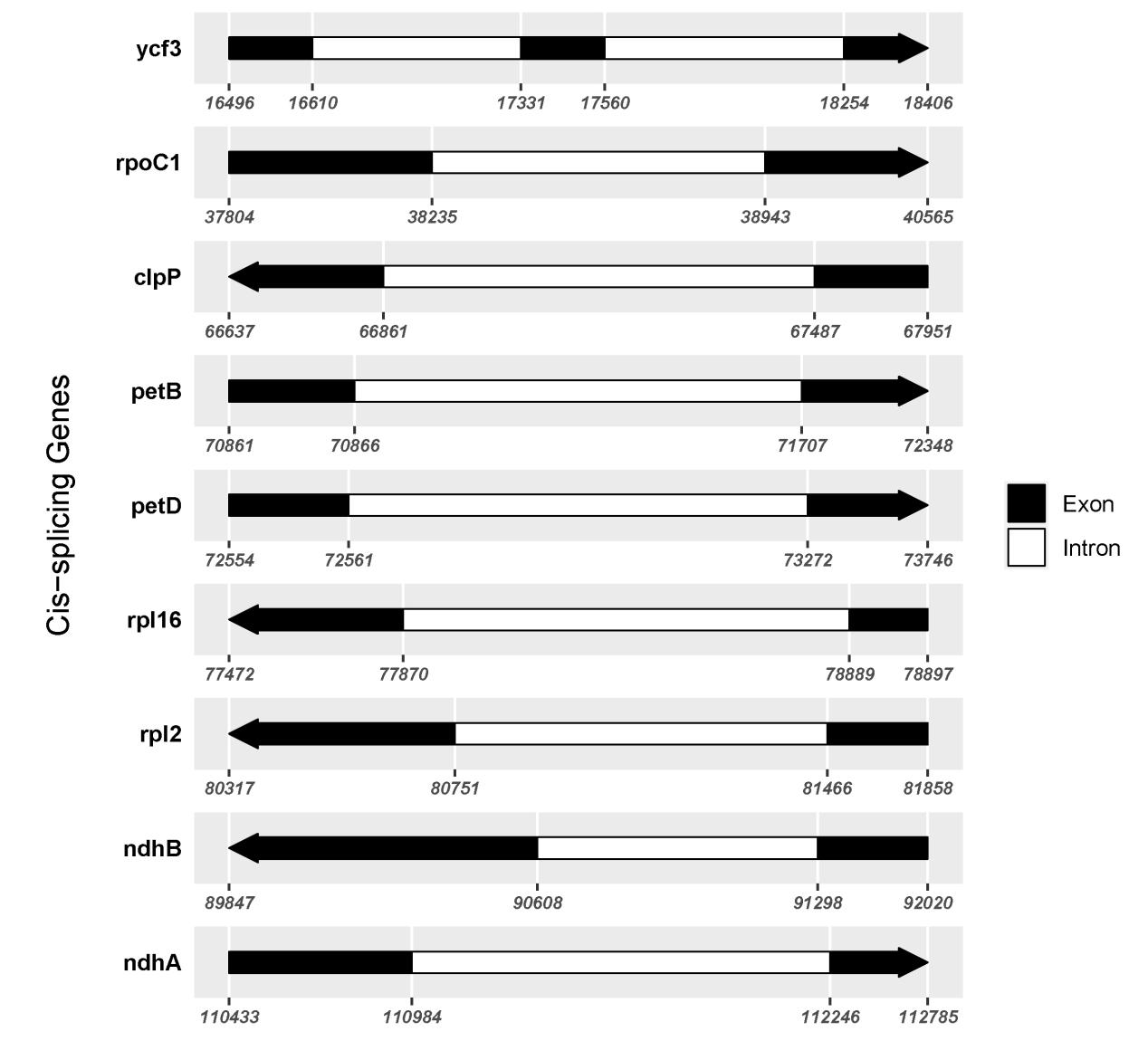
Figure S4 Structure of Cis-splicing genes in the *Oxytropis monophylla*.
